# Giant Vortex Dichroism in Simplified-Chiral-Double-Elliptical Metamaterials

**DOI:** 10.3390/nano15030189

**Published:** 2025-01-25

**Authors:** Shiqi Luo, Kangzhun Peng, Zhi-Yuan Li, Wenyao Liang

**Affiliations:** School of Physics and Optoelectronics, South China University of Technology, Guangzhou 510640, China

**Keywords:** chiral metamaterial, vortex dichroism, orbital angular momentum

## Abstract

Vortex dichroism in chiral metamaterials is of great significance to the study of photoelectric detection, optical communication, and the interaction between light and matter. Here we propose a compact chiral metamaterials structure composed of two elliptical SiO_2_ rods covered with a Au film on a substrate to achieve a significant vortex-dichroism effect. Such a structure has different responses to a Laguerre-Gaussian beam carrying opposite-orbital angular momentum, resulting in giant vortex dichroism. The influences of various structural parameters are analyzed, and the optimal parameters are obtained to realize a remarkable vortex dichroism of about 58.5%. The simplicity and giant VD effect of the proposed metamaterials make it a promising candidate for advancing chiral optical applications such as optical communication and sensing.

## 1. Introduction

Chirality is the property that an object cannot coincide with its mirror image through simple rotational or translational transformations. Chirality is a universal phenomenon in nature [1,2,3], which is widely found in molecular structures, biomacromolecules, structures of living organisms, and even macroscopic objects. In recent years, researchers have been interested in incorporating chirality into the field of optical metamaterials and have been able to fabricate artificial chiral metamaterials (CMMs) using methods such as electron-beam lithography and colloidal lithography [4,5,6,7]. CMMs enable sophisticated control of electromagnetic waves, even at scales comparable to the wavelength of light [8,9].

It is well known that light has energy, momentum, and angular momentum properties. Especially, the angular momentum can be further divided into spin angular momentum (SAM) and orbital angular momentum (OAM), which are related to the polarization state of light and the phase distribution of light, respectively. Circular dichroism (CD) related to SAM is defined as the difference in absorption, reflection, and transmission between right-spin and left-spin circularly polarized light [10,11], and it has a wide range of applications in imaging and polarization detection [12,13]. Compared with SAM, OAM is more commonly used in optical holography, optical communication [14], and super-resolution fluorescence microscopy [15], and it holds great promise in the study of light–matter interactions [16]. Current studies have mainly focused on CD [17], which is related to the photonic SAM, but there have also been a number of studies of OAM conducted in recent years, particularly focusing on chiral light–matter interactions [18,19]. Recent reviews by Forbes and Porfirev have comprehensively summarized the role of OAM in chirality and optical activity, as well as its applications in controlling matter at the micro and nanoscale [20,21].

A vortex beam, also known as an OAM beam, is a special type of beam with a helical phase wavefront that varies along an azimuthal angle, manifesting itself as having a central phase singularity and a circular light intensity distribution. The phase distribution of a vortex beam is described as $e^{il\varphi }$, where the topological charge *l* is an important parameter to characterize the OAM of the beam [22]. The topological charge *l* usually takes integer values, ranging from negative to positive infinity. Specifically, a right-handed wavefront (RHW) or left-handed wavefront (LHW) vortex beam corresponds to the clockwise (−*l*) or counterclockwise (+*l*) rotational direction of the helical phase wavefront, respectively. The electric-field distribution of the primordial optical vortex with opposite topological charges has a mirror-symmetric distribution.

The interaction between vortex beams and CMMs depends on the positive or negative topological charge *l* [23], which means that vortex beams with opposite topological charges can result in different transmission, reflection, and absorption spectra during their interaction with CMMs. Corresponding to CD, this phenomenon is known as vortex dichroism (VD). In 2016, Verbiest’s group first detected that VD resulted from the interaction between a vortex beam and chiral molecules by using a specially formulated nanoparticle polymer to enhance the electric quadrupole field of the chiral molecules [24]. In 2018, Kerber et al. reported the VD phenomenon from the interaction between vortex beam and metallic Archimedean helical structures [25,26]. Subsequently, Fang et al. observed different spectra at different moments by using chiral metal nanoparticles [27]. Forbes’ pioneering work elucidates the interaction between the orbital angular momentum (OAM) of light and chiral particles, particularly how optical vortices influence the optical activity and absorption characteristics of chiral molecules [21,28]. HU et al. used the Richards–Wolf vector-diffraction-integration method to calculate the focal field of radially polarized light carrying OAM after focusing through a high-numerical-aperture objective. The phase structure of radially polarized light carrying OAM would exhibit a helical shape, leading to an 11.9-fold enhancement of the chiral signal in the light field strength compared to circularly polarized light [29]. In recent years, several related experimental studies have been conducted. The OAM carried by the vortex beam can interact with the molecular structure of the chiral tetramer, leading to a change in the reflection or transmission properties of the beam, with the largest VD of about 23% observed [30]. Ni et al. demonstrated gigantic vortical differential scattering as a monochromatic probe for multiscale chiral structures [31]. Additionally, vortex dichroism in disordered molecular media and chiral plasmonic nanoparticles has also been reported [18]. Despite the above progress, the complexity of current CMMs poses technical challenges for practical preparation. In addition, how to further enhance the VD response is also an urgent issue in VD research.

In this work, we propose a compact CMM to achieve a significant VD effect. The designed CMM is composed of two elliptical SiO_2_ rods coated with a Au film on a substrate. The simulation results reveal the influence of various structural parameters, such as the lengths of the major and minor axes, the position, and the rotational angle of the elliptical rod, on the reflectivity of vortex beams with opposite topological charges. The optimal configuration parameters for the CMM are obtained to generate a remarkable VD of about 58.5%. These results indicate that the designed CMM possesses both advantages of a giant VD effect and a compact structure, which holds promise in various VD applications.

## 2. Structure Design

**Figure 1:**
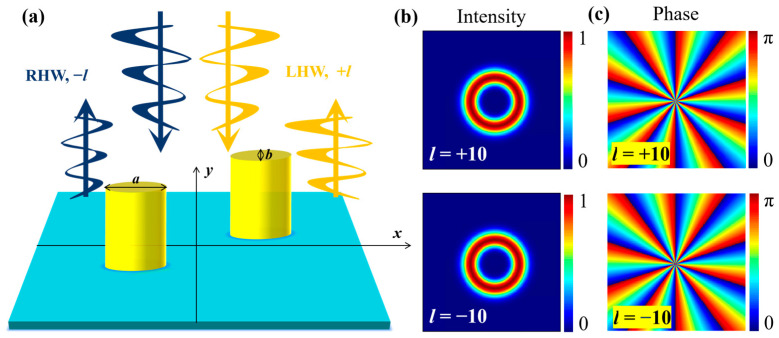
(**a**) Schematic diagram for Laguerre-Gaussian beams with LHW and RHW incident on the designed CMM; (**b**) intensity distributions of the vortex beams with *l* = ±10; and (**c**) phase distributions of the vortex beams with *l* = ±10.

Figure 1a presents the designed CMM composed of two elliptical rods on a substrate, capable of responding to opposite vortex beams and producing strong/weak reflection properties. The two elliptical rods have identical geometrical parameters. The major axis parallel to the *x* direction is a=3.0 μm, while the minor axis *b* is determined by the ratio ρ=b/a=0.5. The height of the rods is *h* = 4 μm. The two elliptical rods are placed at (2.1 μm, 2.1 μm) and (−2.1 μm, −2.1 μm), respectively. The elliptical rods and the substrate are silicon dioxide (SiO_2_). Especially, the two elliptical SiO_2_ rods are coated with a layer of gold (Au) with a thickness of 0.1 μm to enhance the reflectivity. The refractive indices for both SiO_2_ and Au are 1.44 and 0.18 + 5.1 i at 800 nm, which are from the data published by Edward Palik [32]. The proposed structure can be fabricated using direct laser writing for the SiO_2_ pillars, followed by uniform gold coating via ion sputtering, as demonstrated in similar chiral nanostructures [30]. For such CMM, when a RHW (or LHW) vortex beam with −*l* (or +*l*) is incident on it, their reflection behaviors are different, as shown in Figure 1a.

In order to study the VD effect caused by the interaction of a vortex beam with the designed CMM, we employ a Laguerre-Gaussian beam carrying LHW or RHW as the light source, placed 0.5 µm above the CMM, to illuminate the CMM under normal incidence. The Laguerre-Gaussian beam is polarized along the *x* direction, which can avoid the chiral disturbance from the SAM. Under the paraxial approximation, the electric field distribution of the Laguerre-Gaussian vortex beam can be expressed as [30,33],

(1) $${\bar{E}}_{l}^{in}(r,\varphi ,z)=\sqrt{\frac{2}{\pi w^{2}(z)|l|!}}{\left(\frac{\sqrt{2}r}{w(z)}\right)}^{\left|l\right|}\exp(-\frac{r^{2}}{w^{2}(z)})\exp(il\varphi ){\bar{E}}_{x}$$

where *w*(*z*) is the beam radius,

(2) $$w(z)=w_{0}\sqrt{1+{\left(\frac{z}{z_{R}}\right)}^{2}}$$

${\bar{E}}_{x}=E_{x}\hat{x}$ is the horizontal polarization component, and *r*, *φ*, and *z* are the cylindrical coordinates, respectively. In our calculation, the beam waist *w*_0_ is kept constant for all values of *l*, while the overall beam diameter increases with *l*. This ensures that the spatial overlap between the vortex beam and the nanostructure varies with *l*, which is crucial for observing the scale-dependent chirality effects [31]. The intensity distribution of the vortex beam is,

(3) $$I(r,\varphi )=\frac{2}{\pi \omega^{2}|l|!}{\left(\frac{\sqrt{2}r}{\omega }\right)}^{2|l|}\exp(-\frac{2r^{2}}{\omega^{2}})|E_{x}{|}^{2}$$

Therefore, the LHW and RHW vortex beams have the same diameter *d* which increases with increasing topological charge |*l*|. As an example, Figure 1b,c illustrate the distributions of intensities and phases of the Laguerre-Gaussian vortex beams characterized by *l* = +10 and −10. Obviously, their intensity distributions are the same, while their phase distributions are totally inverse, manifesting the fundamental characteristics of LHW and RHW vortex beams carrying opposite topological charges.

## 3. Results

### 3.1. Reflection and VD Analyses

To facilitate a quantitative assessment of VD effect, we adopt a similar approach to that used for CD and define the parameter VD as follows,

(4) $$VD=\frac{R_{+l}-R_{-l}}{\left(R_{+l}+R_{-l}\right)/2}\times 100\%$$

where *R_+l_* and *R_−l_* represent the reflected intensities of the LHW (+*l*) and RHW (−*l*) vortex beams, respectively.

**Figure 2:**
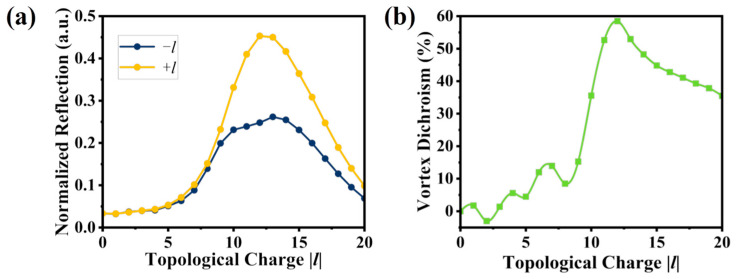
(**a**) Normalized reflection spectra of LHW and RHW for the designed CMM; (**b**) the corresponding VD curve.

We have calculated the normalized reflection spectra at 800 nm after the Laguerre-Gaussian beams carrying LHW and RHW interact with the CMM. All the simulations are carried out using Lumerical FDTD Solutions software based on the Finite Difference Time Domain (FDTD) method. Perfectly matched layers are applied as boundary conditions in ±*x*, ±*y* directions to minimize the reflection from boundaries. Since SiO_2_ is highly transparent at the operating wavelength (800 nm), the SiO_2_ substrate has a negligible impact on the transmittance and reflection. Moreover, we mainly focus on the VD effect resulting from reflection difference. Figure 2a shows the calculated normalized reflection spectra results when the topological charge |*l*| ranges from 0 to 20 under the condition of the same incident intensity. For the case of LHW vortex beam (+*l*), as |*l*| increases from 0 to 20, the reflection firstly increases and then decreases, leading to a maximum value of 0.45 at *l* = 12. Similarly, for the RHW vortex beam (−*l*), the reflection follows a similar trend, reaching a maximum of 0.24 at *l* = 12. It is noted that the two reflection curves exhibit significant differences, i.e., the reflection of the LHW optical vortex (+*l*) is consistently higher than that of the RHW one (−*l*). This indicates that chiral structures can differentiate between vortex beams carrying opposite topological charges. The underlying physical mechanism is attributed to the scale-dependent chirality of vortex light. The scale-dependent chirality refers to the different behaviors exhibited by its chiral properties at different scales, which are closely related to factors such as the phase gradient and topological charge of vortex beams. When the vortex beam interacts with chiral molecules or chiral structures, the reflection characteristics of LHW and RHW appear significantly different. Specifically, with increasing *l*, the beam width $d_{l}=w\sqrt{2|l|}$ increases accordingly. Lower values of *l* result in smaller beam waist, while high values of *l* lead to larger beam waist. As a result, around *l* = 12 is clearly the optimal spatial overlap in our system.

According to Formula (3) and Figure 2a, the VD spectrum for the designed CMM is calculated and shown in Figure 2b. The VD values are always positive for 0 < |*l*| < 20. With increasing |*l*|, the VD value firstly increases slowly (when |*l*| < 10), then ascends steeply to a peak of about 58.5% at |*l*| = 12 and finally descends slowly. Compared to the 20% vortex dichroism reported [31], our structure achieves a significantly higher VD of 58.5%, which is attributed to the optimized geometry and enhanced light–matter interaction. Besides, the oscillatory behavior from *l* = 0 to 9 can be explained as follows. For low values of *l*, the beam width is relatively small, and the spatial overlap between the vortex beam and the chiral structure is not optimal. As a result, the interaction between the vortex beam and the CMM is weak, leading to small difference between the normalized reflections for +*l* and −*l* and thus the observed oscillations.

**Figure 3:**
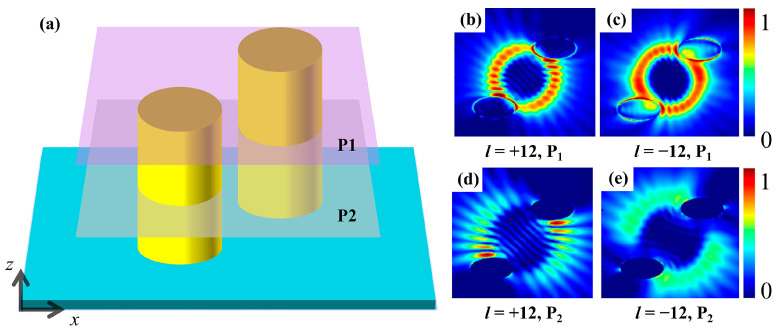
Electric field distributions measured at different locations: (**a**) schematic of the detector positioned within the Au layer, with two detection planes labeled as P_1_ and P_2_; (**b**,**c**) field distributions corresponding to *l* of +12 and −12 at P_1_, respectively; and (**d**,**e**) field distributions corresponding to *l* of +12 and −12 at P_2_, respectively.

To reveal the origin of asymmetric transmission of vortex beams with opposite topological charges on the CMM, the process of light–matter interaction needs to be analyzed. We take the maximum VD case in Figure 2b with *l* = ±12 as an example for further discussion. Two reflection monitor planes are placed at the top and the middle of the elliptical rods, as depicted by planes P_1_ and P_2_ in Figure 3a, respectively. Figure 3b,c display the electric field distributions for *l* = +12 and −12, respectively, as measured at plane P_1_. It is shown that when the vortex beam first contacts the CMM, the difference in the electric field distributions is not significant. Similarly, Figure 3d,e present the electric field distributions for the same *l* = +12 and −12, but measured at plane P_2_. After the beam has fully interacted, the electric field distributions of the vortex beams with opposite *l* exhibit a noticeable difference. The distinct interaction emerges for vortex beams with opposite *l* with CMM due to phase inconsistencies, leading to different reflection signatures observed at P_2_ and producing the VD effect.

### 3.2. Structure Optimization for Giant VD

**Figure 4:**
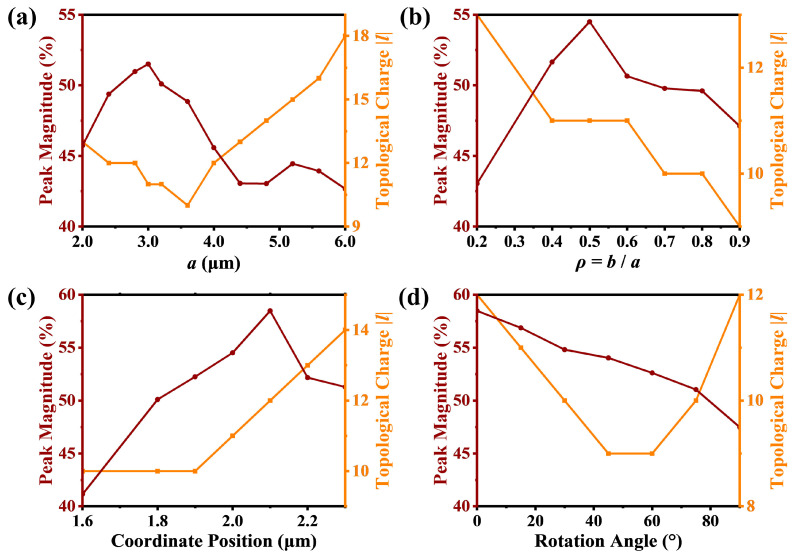
VD peak curve (red) and the corresponding topological charge curve |*l*| (orange) obtained by adjusting different geometric parameters. (**a**) The case of changing major axis of the elliptical rod; (**b**) the case of changing *ρ*; (**c**) the case of changing the coordinate positions of the two elliptical rods; and (**d**) the case of changing the rotation angle.

To acquire the optimal VD, we have carried out parameter optimization of the designed CMM. The reflectivity is recorded by a monitor positioned 0.5 um above the incident light source whose wavelength is 800 nm. Figure 4 presents the calculated peak magnitudes of VD when *a*, *ρ*, coordinate position or rotation angle of the elliptical rods varies independently. The initial parameters are *a* = 3.0 μm, *ρ* = 0.4, coordinate positions at (2.0 μm, 2.0 μm) and (−2.0 μm, −2.0 μm), and a rotation angle of 0°, respectively. It should be noted that the left vertical axis shows the peak magnitude, while the right vertical axis denotes the topological charge |*l|*.

Firstly, we discuss the influence of *a* and *ρ* on the VD effect. As shown in Figure 4a, the VD peak curve has a maximum of approximately 52% at *a* = 3.0 μm, corresponding to |*l*| = 11. Subsequently, we study the VD peak when *ρ* increases from 0.2 to 0.9. Figure 4b reveals that the VD reaches a maximum of about 55% when *ρ* = 0.5 and |*l|* = 11. Based on Figure 4a,b, one can get the optimal parameters of *a* = 3.0 μm and *ρ* = 0.5 to generate a significant VD effect, which will be adopted in the following discussion.

Secondly, we further discuss the influence of the position of the elliptical rods on the VD peak. The positions of the elliptical rods denoted by (*x*, *x*) and (−*x*, −*x*) are systematically varied by simply changing the value of *x* from 1.6 to 2.3, as shown in Figure 4c. The results show that there exists a maximum of 58.5% for the VD peak curve at positions (±2.1 μm, ±2.1 μm), corresponding to |*l|* = 12. This maximum VD peak exceeds the results in Figure 4a (52%) and 4b (55%). It is noted that as *x* > 1.9 μm, the distance between the two elliptical rods becomes much larger, which requires a vortex beam with a bigger diameter (i.e., larger |*l*|) to overlap with each other better to generate a pronounced VD effect.

Finally, we investigate the impact of orientation of the elliptical rods on the VD peak. The rotational angle is defined as the angle between the major axis of the elliptical rod and the *x*-axis. Figure 4d presents the VD peak curve as the rotational angle increases from 0° to 90°. Surprisingly, the VD peak is most pronounced at 0° where the major axis is parallel to the *x*-axis, and it does not increase further at greater angles. This indicates that the rods’ initial orientation is most conducive to maximizing VD. As the rotational angle increases, the VD peak appears at |*l*| = 12 for 0°, then descends to |*l*| = 9 for 45°, and finally returns to |*l*| = 12 for 90°. The identical |*l*| values at 0° and 90° further suggests that the intensity distribution of the vortex beam is consistent. However, the resulting VD values are different, which indicates that the phase of the vortex beam is an important factor to affect the reflectivity as well as the VD effect.

In summary, based on the above discussions about structure optimization, it is found that the geometric parameters for the designed CMM play important roles in the VD effect. The optimal parameters are *a* = 3.0 μm, *ρ* = 0.5, positions of (2.1 μm, 2.1 μm) and (−2.1 μm, −2.1 μm), together with a rotational angle of 0°, which are actually adopted in Figure 1 and Figure 2.

**Figure 5:**
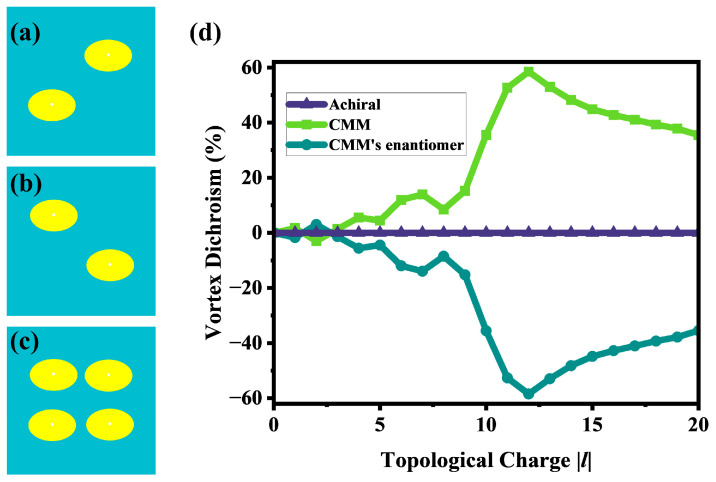
(**a**) Top view of designed CMM; (**b**) top view of the enantiomer of designed CMM; (**c**) top view of achiral metamaterials; and (**d**) VD response of three metamaterials.

Additionally, for the sake of comprehensiveness, we extended our investigation to the enantiomer of the designed CMM (Figure 5b) and an achiral structure (Figure 5c). The calculated results in Figure 5d clearly show that the VD curves for the designed CMM and its enantiomer are completely symmetrical, with the optimal VD value of ±58.5%, indicating that they have opposite reflection characteristics for LHW and RHW vortex beams, respectively. The result for the achiral structure (Figure 5c) is also presented in Figure 5d. It reveals that the absence of chirality leads to VD values approaching 0% for both LHW and RHW vortex beams with ±*l*. Consequently, the VD effect for the achiral metamaterials is negligible, indicating that the giant VD in CMMs is inherently linked to chiral properties.

## 4. Conclusions

In conclusion, we propose a compact double-elliptical-rod structure that exhibits a significant interaction with vortex beams, resulting in a pronounced VD effect. The optimal geometric parameters of the metamaterials for maximum VD of 58.5% are obtained to be a major axis *a* = 3 μm, the ratio *ρ* = 0.5, and elliptical rod positions of (2.1 μm, 2.1 μm) and (−2.1 μm, −2.1 μm), and a rotational angle of 0°. Additionally, it is found that the enantiomer of the designed CMM has total opposite VD values compared with those of the CMM. These results provide a new way to achieve robust chiral responses, which hold potential in various VD applications such as vortex sensors.

## Data Availability

The data presented in this study are available on request from the corresponding author.
